# Case report: Cardiac metastatic leiomyoma in an Asian female

**DOI:** 10.3389/fsurg.2022.991558

**Published:** 2022-08-23

**Authors:** Juan Li, Hong Zhu, Shuang-Ye Hu, Shang-Qing Ren, Xing-Lan Li

**Affiliations:** ^1^ Department of Pathology, Sichuan Provincial People's Hospital, University of Electronic Science and Technology of China, Chengdu, China; ^2^ Chinese Academy of Sciences Sichuan Translational Medicine Research Hospital, Chengdu, China; ^3^ Department of Pathology, Longquanyi District of Chengdu Maternity and Child Health Care Hospital, Chengdu, China; ^4^ Department of Robotic Minimally Invasive Surgery Center, Sichuan Provincial People's Hospital, University of Electronic Science and Technology of China, Chengdu, China

**Keywords:** metastasizing leiomyoma, heart, case report, surgery, outcome

## Abstract

**Background:**

Uterine leiomyomas are the most common gynecological tumors in women of child-bearing age and premenopausal women, while benign metastasizing leiomyomas of the heart are rare.

**Case presentation:**

We report a rare case of metastasizing leiomyoma in the heart of a 54-year-old woman 10 years after a uterine leiomyoma was discovered during hysterectomy. Echocardiography, cardiac plain scan and enhanced MRI at presentation showed a soft tissue signal mass in the right ventricle. A large cardiac mass attached to the chordae of the tricuspid valve and later shown to be histopathologically consistent with uterine leiomyoma was successfully resected through a right atriotomy.

**Conclusions:**

Our case report highlights a rare type of tumor of the heart and suggests that metastasizing leiomyoma should be considered in the differential diagnosis of right-sided cardiac tumors. The complete surgical resection of the tumor was considered to be the best treatment.

## Introduction

Primary cardiac tumors are rare, and most cardiac tumors are secondary to metastatic disease (1). Overall, smooth muscle tumors of the heart are extremely rare. Most of the smooth muscle tumors are occurring in reproductive women who have a history of uterine leiomyoma resection or hysterectomy (2). The presenting symptoms of cardiac tumor depend on the size, location of the mass, and eventual obstruction to the inflow or outflow tracts. Most cardiac tumors are asymptomatic, and diagnosis is made on the basis of heart murmur, arrythmias. Echocardiography, cardiac magnetic resonance imaging (MRI), and computed tomography (CT) usually are useful for establishing the diagnosis, although pathological diagnosis remains the gold standard for diagnostic confirmation (3). Here, we report a rare case of metastasis of uterine leiomyoma to the heart. The aim of this report is to increase awareness of this metastatic tumor. This study was reported in agreement with principles of the CARE guidelines (4).

## Case presentation

**Figure 1:**
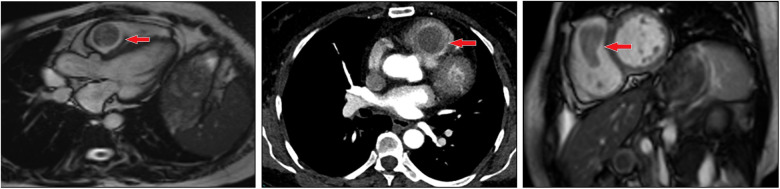
Imaging of metastasizing leiomyoma. Enhanced MRI revealed soft tissue signal mass shadows in the outflow tract of the right ventricle.

A 54-year-old woman presented to the hospital due to with a heart murmur detected at a routine examination. On physical examination, the patient's general condition and vital signs were quite unremarkable; only the 2nd to 3rd costal segment of the left margin of the sternum appeared abnormal, and 3/6 systolic blow-like murmurs could be heard; pericardial frictional sounds were not heard, and there was no edema in either lower limb. Echocardiography indicated right ventricular space occupation. Cardiac plain scan and enhanced MRI showed soft tissue signal mass shadows in the outflow tract of the right ventricle (Figure 1). CT angiography showed no macrovascular lesions. What's more is that patient had undergone hysterectomy in a local hospital 10 years previously due to excessive vaginal bleeding from uterine leiomyoma. The family histories were noncontributory to the diagnosis.

The patient underwent right ventricular mass resection under general anesthesia with low-temperature extracorporeal circulation. The patient recovered well after the surgery. The surgical findings showed that there was a 3 cm × 3 cm oblong tomato-like mass with a smooth surface and tough texture occupying the right ventricle. The tumor pedicle was connected to the chordae of the tricuspid valve and protruding into the right ventricular outflow tract, but other portions of the tumor had no adhesion to the heart. The specimens were laid out along their longitudinal axes and fixed in 10% neutral formalin for 12 h. Ten representative tissue samples were taken from different areas, dehydrated, embedded in paraffin, and sequentially sectioned into 3-μm-thick sections. The sections were incubated with primary antibodies against desmin, ER, PR, myogenin, MyoD1, caldesmon, pan-CK, Ki-67, SMA, CD117, FH, RB-1, MDM2, and CD34. PBS replaced the primary antibody as a negative control. DAB color development and hematoxylin counterstaining were performed. The primary antibodies and the kit were purchased from Beijing Zhongshan Jinqiao Company.

**Figure 2:**
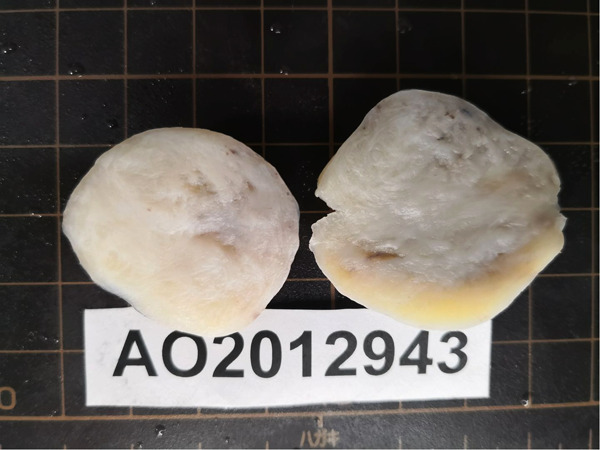
Macropathology of metastasizing leiomyoma. The leiomyoma appeared as a solid, tough, grayish-white tubercle with no exact capsule.

**Figure 3:**
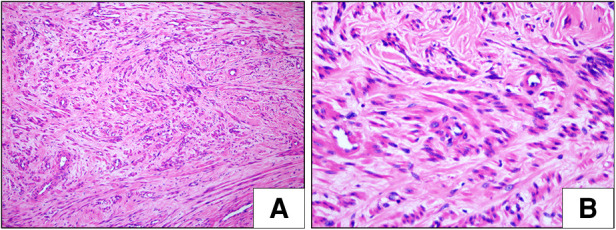
Histological examination (HE) of metastasizing leiomyoma. (**A,B**) The spindle cell tumor showed clear boundaries, mild cell morphology, fascicular arrangement, and no mitotic figures (H&E staining, **A**: ×100, **B**: ×400).

**Figure 4:**
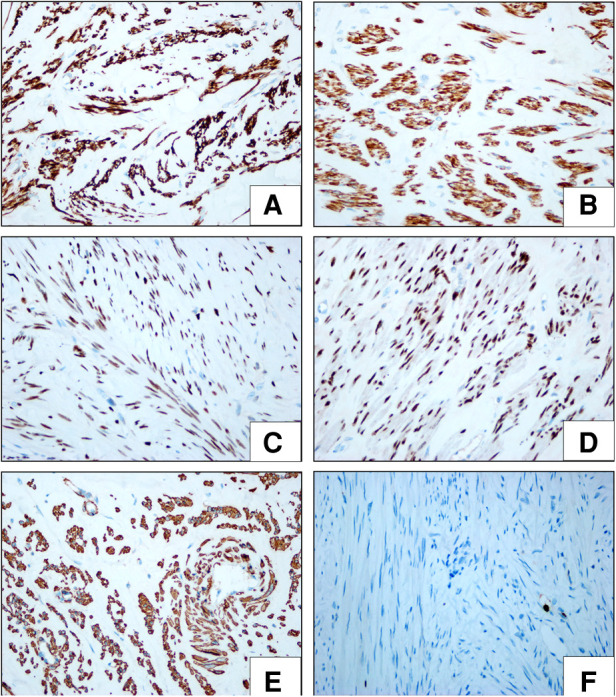
Immunohistochemical (IHC) features of metastasizing leiomyoma. (Envision × 400) The spindle cells expressed desmin (**A**), caldesmon (**B**), PR (**C**), ER (**D**) and SMA (**E**). The calculated Ki67 labeling index was less than approximately 2% (**F**).

On histopathologic examination, the tumor appeared as a grayish-white tubercle, with no exact capsule; it was solid and tough and measured 3 cm × 3 cm × 1.5 cm in size (Figure 2). Under the microscope, the spindle cells within the tumor showed clear boundaries, mild cell morphology, fascicular arrangement, and no mitotic figures (Figure 3). Immunohistochemical staining for desmin, caldesmon, ER, PR and SMA was positive, and the Ki-67 index was approximately 2% (Figure 4). These morphologic features and the immunohistochemical staining pattern confirmed a diagnosis of benign metastasizing leiomyoma to the heart.

## Discussion

### Case review

Benign metastasizing leiomyoma is very rare. Benign metastasizing leiomyoma involving the heart was first reported by Timmis in 1980, and at least 8 other cases were subsequently reported (Table 1).

**Table 1:** Summary of case reports of metastasizing leiomyoma in the heart.

Patient age/gender	Metastasis site(s)	Cardiac tumor size	Interval to metastasis	Location in heart	References
46/F	Heart	7.5	1.5 years after hysterectomy	Inferior vena cava	Timmis AD, et al. (1980) (5)
44/F	Heart, lung	4.7	4 years after hysterectomy	Anterior papillary muscle of right ventricle	Takemura G, et al. (1996) (6)
41/F	Heart	3.5	3 months after hysterectomy	Right anterior interventricular septum	Galvin SD, et al. (2010) (1)
37/F	Heart, lung, soft tissue, liver and enterocoelia	NG*	11 years after leiomyoma resection	Right cardiac chamber and cardiac wall	Cai A, et al. (2014) (7)
55/F	Heart, lung	4	16 years after hysterectomy	Right atrium	Consamus EN, et al. (2014) (8)
51/F	Heart, lung	4.5	Simultaneously	Right ventricle	Williams M, et al. (2016) (9)
36/F	Heart, lung	4.7	12 years after hysterectomy	Abutted the pulmonary valve	Meddeb M, et al. (2018) (10)
46/F	Heart, lung and Pelvic	4.8	10 years after hysterectomy	The tricuspid valve	Mohamed M, et al. (2020) (11)
45/F	Heart, lung	9	2 years after hysterectomy	The tricuspid valve	Karnib M, et al. (2021) (12)
54/F	Heart	5	10 years after hysterectomy	The tricuspid valve	Current case

* NG, not given.

### Clinical manifestations, diagnosis, treatment and prognosis

Metastasizing leiomyomas of the heart are most common in middle-aged women. Most patients with metastasizing leiomyoma have a history of hysterectomy/myomectomy combined with an existing hysteromyoma. In the case described here, the patient had a history of hysteromyoma resection, and the time from the first hysteromyoma resection to the discovery of cardiac leiomyoma was 10 years. The clinical symptoms of metastasizing leiomyoma are nonspecific; the most common symptoms are dyspnea, syncope, edema of the lower extremities, and palpitations. The patient described here had no clinical symptoms, and the leiomyoma was found by chance during physical examination. TEE, CT and MRI can effectively assist preoperative diagnosis and guide surgical treatment. Although clinical manifestations and imaging can provide early and accurate assessment of the disease, the final diagnosis depends on postoperative pathological examination. Patients in whom there was complete tumor resection had a good prognosis, and no postoperative recurrence or death was reported. Postoperative recurrence is common in patients with incomplete tumor resection. In cases in which the tumor cannot be completely removed, some researchers have suggested the use of antiestrogen drugs (including tamoxifen and letrozole) after surgery. Awonuga (13) and Rivera (14) found that the tumor subsided after the use of antiestrogen drugs after surgery. In our case, the patient underwent right ventricular mass resection by surgery, and have not received any medication. Postoperative cardiac MRI indicated that the mass was completely removed, and there was no recurrence after 12 months follow-up.

### Pathological etiology

The behavior of cardiac leiomyoma is benign. Microscopically, the tumor in our patient was composed of fasciculate spindle smooth muscle cells with scattered stroma in small blood vessels. No obvious bleeding or necrosis was observed, and nuclear atypia, pleomorphism and mitoses were also rare. The rare types of pathological variation previously reported include angiomyoma (15), adipose leiomyoma (16), lymphangiomyoma (17) and borderline leiomyoma (18). In our case, immunohistochemistry showed positive results for smooth muscle markers such as SMA and desmin, and the lack of expression of vimentin ruled out a myofibroblastic nature of the tumor. Intrauterine tumors have histopathological and immunohistochemical characteristics that are the same as those of cardiac leiomyomas originating in the uterus. Some researchers have conducted molecular genetic analysis of cardiac leiomyoma; the results of those studies showed that almost all of the analyzed samples had abnormal genotypes such as 45, XX, Der (14) T (12;14) (Q15; Q24) (19), However, as cardiac leiomyomas are clinically rare, their etiology remains to be further studied.

### Histological origin

At present, there are two views on the histological occurrence of leiomyomas. One is that the tumor originates from uterine fibroids and extends into veins; this is the case for the metastasizing leiomyomas reported in most studies (20–22). The second is that primary cardiac leiomyomas may originate from the smooth muscle tissue of the vein wall. In this case, the tumor extends upward along the venous system and may exceed the inferior vena cava and enter the right atrium, right ventricle and even the pulmonary artery (15). In our case, medical history, cardiac imaging findings and the immunohistochemical staining pattern confirmed a diagnosis of benign metastasizing leiomyoma originated from uterine fibroids.

### Differential diagnosis

The differential diagnosis of cardiac leiomyoma mainly includes cardiac myxoma, cardiac leiomyosarcoma, and other metastasizing cardiac tumors. Cardiac myxoma mostly occurs in the left cardiac system, with an incidence of 87.3% to 85.5%; in this respect, it differs from cardiac leiomyoma, which only affects the right cardiac system (23–25). Most atrial myxomas are pedicled to the atrial septum or wall and may be adherent to the wall. Histological morphology, preoperative imaging examination and history of uterine leiomyoma are helpful for differential diagnosis. Leiomyosarcomas in the heart are malignant tumors with similar clinical and imaging manifestations as leiomyomas of the heart, and the two need to be differentiated through postoperative pathology and immunohistochemical diagnosis. Moreover, leiomyosarcomas have the behavioral characteristics of malignant tumors and often metastasize to other organs or sites such as the lung, lymph nodes, bone, skeletal muscle, subcutaneous tissue and retroperitoneal space but do not extend and grow in the venous system (26). Other metastasizing cardiac neoplasms often invade the pericardium or myocardial tissue, and their primary foci can be determined by systemic radionuclide scanning.

## Conclusion

Although metastasizing leiomyoma is rare, this tumor should be considered in the differential diagnosis of cardiac tumors, especially in patients with a history of uterine fibroids. Future research is needed to better understand the pathogenesis and treatment of such tumors.

## Data Availability

The raw data supporting the conclusions of this article will be made available by the authors, without undue reservation.
